# Updating test-day milk yield factors for use in genetic evaluations and dairy production systems: a comprehensive review

**DOI:** 10.3389/fgene.2023.1298114

**Published:** 2023-12-11

**Authors:** Xiao-Lin Wu, George R. Wiggans, H. Duan Norman, Malia J. Caputo, Asha M. Miles, Curtis P. Van Tassell, Ransom L. Baldwin, Steven Sievert, Jay Mattison, Javier Burchard, João Dürr

**Affiliations:** ^1^ Council on Dairy Cattle Breeding, Bowie, MD, United States; ^2^ Department of Animal and Dairy Sciences, University of Wisconsin, Madison, WI, United States; ^3^ USDA Animal Genomics and Improvement Laboratory, Beltsville, MD, United States; ^4^ National Dairy Herd Information Association, Verona, WI, United States

**Keywords:** dairy cattle, genetic evaluation, lactation curve, non-linear models, test-day yields

## Abstract

Various methods have been proposed to estimate daily yield from partial yields, primarily to deal with unequal milking intervals. This paper offers an exhaustive review of daily milk yields, the foundation of lactation records. Seminal advancements in the late 20th century concentrated on two main adjustment metrics: additive additive correction factors (ACF) and multiplicative correction factors (MCF). An ACF model provides additive adjustments to two times AM or PM milk yield, which then becomes the estimated daily yields, whereas an MCF is a ratio of daily yield to the yield from a single milking. Recent studies highlight the potential of alternative approaches, such as exponential regression and other nonlinear models. Biologically, milk secretion rates are not linear throughout the entire milking interval, influenced by the internal mammary gland pressure. Consequently, nonlinear models are appealing for estimating daily milk yields as well. MCFs and ACFs are typically determined for discrete milking interval classes. Nonetheless, large discrete intervals can introduce systematic biases. A universal solution for deriving continuous correction factors has been proposed, ensuring reduced bias and enhanced daily milk yield estimation accuracy. When leveraging test-day milk yields for genetic evaluations in dairy cattle, two predominant statistical models are employed: lactation and test-day yield models. A lactation model capitalizes on the high heritability of total lactation yields, aligning closely with dairy producers’ needs because the total amount of milk production in a lactation directly determines farm revenue. However, a lactation yield model without harnessing all test-day records may ignore vital data about the shapes of lactation curves needed for informed breeding decisions. In contrast, a test-day model emphasizes individual test-day data, accommodating various intervals and recording plans and allowing the estimation of environmental effects on specific test days. In the United States, the patenting of test-day models in 1993 used to restrict the use of test-day models to regional and unofficial evaluations by the patent holders. Estimated test-day milk yields have been used as if they were accurate depictions of actual milk yields, neglecting possible estimation errors. Its potential consequences on subsequent genetic evaluations have not been sufficiently addressed. Moving forward, there are still numerous questions and challenges in this domain.

## Introduction

Accurate lactation records play an indispensable role in the genetic advancement and comprehensive management of dairy cattle, with test-day yields constituting the core of these records. In countries such as the United States, most cows participating in milk recording programs have their milk sampled and milk weights documented monthly (Voelker, 1981). The term “test-day milk yield” thus defines the milk quantity produced by a dairy cow on the specific day when her yield is assessed. Test-day milk yields are used subsequently to predict lactation milk yields (VanRaden, 1997; Cole et al., 2009). Analyzing milk samples provides further information about components, including fat, protein, and somatic cell count. For effective herd management, periodic test-day records serve as a significant source of information about the productivity of individual cows and the overall herd (Everett et al., 1994). Such information is also routinely utilized to assess the cow’s health and milk quality and, in some instances, help determine milk pricing. For genetic evaluations, test-day records are used to calculate estimated breeding values (**EBV**s) for traits related to milk production (e.g., Jamrozik et al., 1997a; VanRaden et al., 2014). The latter information is instrumental in making breeding decisions to improve the herd’s genetic potential for these traits for future generations (Powell and Norman, 2006).

The frequency of test-day recordings can vary depending on the herd management strategies employed. While a cow is milked multiple times on a test day, not all these milkings are weighed. This practice, emerging in the United States in the 1960s, reflected the shift from the standard supervised twice-daily, monthly testing scheme towards cost-efficient milking plans to minimize costs associated with DHIA supervisor visits (Putnam and Gilmore, 1968). Plans such as the AM-PM method alternate sampling during morning or evening milkings across lactation. Initially, each test-day milk yield was taken to be twice a single yield, assuming that morning and evening milking sessions are equally spaced, each spanning precisely 12 h. Or, the biases will cancel out if the unevenness is complementary between AM and PM milkings. However, the practical situations are different. The AM and PM milking intervals may differ, and the rates of milk secretion can fluctuate between daytime and nighttime. AM milking intervals are typically more extended than PM milking intervals, and AM milk yields usually surpass PM milk yields (Putnam and Gilmore, 1970). While the differences are present, they do not necessarily cancel out. Wu et al. (2022) showed a broader range for morning milking intervals (from 5.6 to 23.67 h) compared to evening milking intervals (from 5.0 to 18.4 h), based on a US Holstein dairy cattle dataset comprising 7,544 milking records from 5,201 Holstein cows across 23 herds. The average morning and evening milking intervals were 12.3 and 11.6 h, respectively. Coinciding with the extended morning milking interval, the mean morning milk yield (16.4 kg) exceeded the mean evening milk yield (15.3 kg). Further, a generalized additive model applied to the same dataset indicated that an average US Holstein cow had a higher probability (63.0%) of producing more milk in the morning milking compared to the evening milking, primarily due to more extended AM milking intervals, whereas the reverse probability favoring a larger evening milk yield was only 35.8% (Wu et al., 2023b).

**FIGURE 1:**
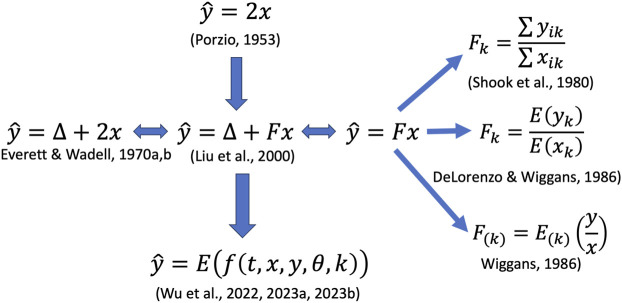
Illustration of various methods proposed for estimating daily milk yields. Notations: x represents a partial (morning or evening) milk yield; $\hat{y}$ stands for an estimated test-day milk yield; ∆ (F) is an additive (multiplicative) correction factor for each milking interval class; j indexes morning ( j=AM ) or evening ( j=PM ) milking, k indexes milking interval classes; E represents expectation operation; f stands for a non-linear function; t is a random variable for milking interval; θ collectively include all variables other than t , x y , and *k*.

A plethora of methodologies has been proposed to address biases in daily yield computations, primarily arising from unequal milking intervals (Figure 1). Central to these advancements, from the 1980s to the 1990s, were the additive correction factors (**ACF**s) and multiplicative correction factors (**MCF**s). For instance, with AM-PM milking, an ACF model supplies additional adjustment quantities over twice the AM (or PM) yields to estimate the daily totals. In contrast, an MCF is defined as a ratio of daily yield to the yield from a single milking, hence also referred to as a ratio factor. Both ACFs and MCFs are computed for discrete milking interval classes. MCFs have been proposed in multiple forms (e.g., Shook et al., 1980; DeLorenzo and Wiggans, 1986), and their exact statistical interpretations differ (Wu X-L. et al., 2023). Wu et al. (2023a) argued that the MCF models encounter a particular challenge, termed ‘the ratio problem’, due to the use of a ratio variable (i.e., proportional daily yield) as the dependent variable in the data density (Wiggans, 1986) or the smoothing functions (Shook et al., 1980; DeLorenzo and Wiggans, 1986). The potential ramifications of this issue could lead to biases in two main areas: the bias from omitted variables and the bias from measurement errors (Lien et al., 2017). In response to ‘the ratio problem’, Wu et al. (2022), Wu et al. (2023b) further proposed an alternative solution in the form of an exponential regression model, which demonstrated improved accuracy for estimating test-day milk yields. Wu et al. (2003b) also evaluated non-linear modeling strategies that relax the linearity assumption of the Wiggans (1986) model in a US Holstein milking dataset. Their results demonstrated that some non-linear models, such as cubic splines, LOESS (locally estimated scatterplot smoothing), and generalized additive models (GAM), were promising as they enhanced the accuracy of estimated daily milk yields. Particularly, GAM provides a flexible tool to capture non-linear relationships in the data by utilizing different non-linear functions for different predictor variables. GAM, when optimally constructed, had the smallest errors and highest accuracies for estimating daily milk yields among all the non-linear models evaluated (Wu et al., 2023b).

The past 2 decades have witnessed a surge in genomic selection studies focusing mainly on genotypes and statistical paradigms. Although lactation records are equally significant, they have been overlooked. The current systems for lactation records and adjustments are somewhat outdated. Recent endeavors for a large-scale dairy data collection, backed jointly by the US Council on Dairy Cattle Breeding, the USDA Animal Genomics and Improvement Laboratory, and the National Dairy Herd Information Association, are converging toward updating the parameters for the current lactation recording systems, assessing existing methods, and exploring new methodologies for producing more reliable lactation records. This paper offers an exhaustive review of daily milk yields, the foundation of lactation records. We aim to provide readers with a multifaceted and in-depth understanding of test-day milk yields, emphasizing daily yield correction factors for estimating daily milk yields and their implications for genetic evaluations. While test-day yields bear immense significance for dairy management, this review does not delve into its expansive scope. Nonetheless, we aspire that this technical overview enlightens a broad spectrum of stakeholders in the dairy sector, encompassing dairy farmers, geneticists, animal scientists, and developers of dairy technology.

## The roadmap

### Primary landmarks

The “AM-PM milking” was the standard practice in dairy farming, whereby cows were milked twice daily: once in the morning (AM) and once in the evening (PM). Originating from the era when manual milking was ubiquitous, this regimen facilitated convenience by allowing cows to be milked in the mornings and evenings (Porzio, 1953; Putnam and Gilmore, 1968). The protocol for each milking session is similar: cows are brought in from the pasture or housing, and their udders and teats are cleaned. Then, the milking process commences, either mechanically or manually. After milking, cows are fed and returned to their resting areas. The AM-PM milking plan not only promotes the cow’s comfort by avoiding overfilling its udder, thus lowering the chance of cow distress and some possible health problems, but also fosters the milking consistency that a cow responds positively to. Additionally, it allows the farmers to monitor the cows for any health and behavior anomalies regularly.

Technological advancements, notably automatic (robotic) milking systems, have ushered in an era where thrice daily milking (3X) or even more frequent sessions are becoming common. Such systems permit more frequent and voluntary milking, potentially elevating milk yield and augmenting animal welfare (Barnes et al., 1990; Erdman and Varner, 1995; Smith et al., 2002; Hart et al., 2013). From a biological standpoint, more frequent milking entails more frequent udder emptying, potentially stimulating heightened milk production. However, these more frequent milkings are not without challenges because they demand more labor, potentially increase cow stress if mismanaged, and necessitate additional diet modifications to cater to increased nutritional needs for augmented milk production. Studies have indicated that while milk yield might rise, milk fat and protein contents could otherwise decline (Barnes et al., 1990; Erdman and Varner, 1995; Smith et al., 2002; Hart et al., 2013). A 2014 USDA report revealed that roughly 88% of dairy operations still predominantly milked cows twice daily, a trend especially prevalent in very small (<30 heads), small (30–99 heads), and medium operations (100–499 heads), with 84.4%–97.9% milking most cows twice daily. In contrast, around 57% of large operations opted for a three-times daily milking schedule (https://www.aphis.usda.gov/animal_health/nahms/dairy/ downloads/dairy14/Dairy14_dr_PartI_1.pdf).

In the 1960s, cost-saving milking strategies emerged wherein not all milkings on a test day were weighed. For instance, in the AM-PM plan, AM and PM milkings were alternately measured on test days throughout lactation. Initially, the daily milk yield was determined as double the amount of a single sampled milking, mathematically represented as (Figure 1):

$$\hat{y}=2x$$ (1)

where x denotes the known AM or PM yield for a cow, and $\hat{y}$ is an estimated daily milk yield. This naïve approach, referred to as the ‘baseline model’ or “2X” method in this paper, relies on the assumption that the intervals and yields of AM and PM milkings are identical, and so are AM and PM milk yields. Therefore, it unexceptionally assigns a fixed multiplicative correction factor for all cows. However, in reality, AM and PM milking intervals are often not equal, and neither are AM and PM milk yields.

An original ACF model is essentially a factorial (or analysis of variance) model that evaluates the difference between AM and PM yield as the response variable. The factorial variables or predictors include milking interval classes, lactation months, and more as appropriate (Everett R. W. and Wadell L. H., 1970; Everett R. W. and Wadell L. H., 1970). Wu et al. (2022), Wu X-L. et al. (2023) have shown that an ACF model can be interpreted as a linear regression of test-day milk yield on the partial yield, milking interval, and other covariates or factors as the predictor variables where applicable. Consider milking interval time (*t*) as the only predictor variable for simplicity. Let *x* be a PM yield and y be the daily total. Then, (*y*-*x*) quantifies the AM yield. The ACF model can then be expressed as follows:

$$E\left\{\left(y-x\right)-x\right\}=\alpha +\beta t$$ (2)

where α and β are the intercept and regression coefficients for milking interval time *t,* respectively. If AM and PM milkings are to be analyzed jointly, the above model can be set up to include heterogeneous intercepts and common slopes for AM and PM (Wu et al., 2022; Wu et al., 2023 X-L.). After some re-arrangement, model (2) becomes:

$$E\left(y\right)=∆+2x$$ (3)

where ∆=α+βt is the additive correction term. Here, the regression coefficient for the partial yield is precisely two, meaning that the additive correction term is supplemented to twice a partial, which then becomes the estimated total daily yield. Setting Δ to zero reverts an ACF model back to the baseline model. ACF are calculated for discrete milking interval classes.

Multiplicative correction factors are ratios of daily yield to yield from single milkings, denoted as F , also computed for discrete milking interval classes (e.g., Shook et al., 1980; DeLorenzo and Wiggans, 1986; Wiggans, 1986) (Figure 1). MCF models have been proposed in multiple forms (Wu et al., 2022; Wu X-L. et al., 2023). The two most common approaches include the DeLorenzo and Wiggans (1986) model and the Wiggans (1986) model. The former linearly regressed test-day yields on a partial yield without intercept daily, whereas the latter treated proportional daily yield as a linear function of milking interval time. A total daily yield is then estimated by *F* times a single yield, where its value varies with different milking interval classes and between AM and PM milkings. Letting F=2 reverts the MCF model back to the baseline model.

Liu et al. (2000) reviewed various linear (and quadratic regression) models where the dependent variable was test-day milk yields. For instance, the linear model is mathematically represented as (Figure 1):

$$\hat{y}=∆+Fx$$ (4)

where ∆ ( F ) is the additive (multiplicative) correction factor calculated for each milking interval class. Seemingly, the linear model accommodates both scenarios, but, effectively, it resembled more ACF models than MCF models (Wu et al., 2022; Wu X-L. et al., 2023). Setting F to 2 in (4) results in an ACF model, whereas letting ∆ equal 0 leads to an MCF model (DeLorenzo and Wiggans, 1986). When both constraints are in place (i.e., ∆=0 and F=2 ), Equation 4 simplifies to be the baseline model.

Here, we illustrate the methods using AM-PM milking as an example. Yet, these same principles can be applied to three times or even more frequent milkings. We also presume that unequal milking interval represents the only source of variation for daily milk yields to simplify the discussion at this stage. Nevertheless, incorporating more affecting variables or factors, such as DIM, into the model is feasible and straightforward.

### Exponential regression model

The exponential regression model proposed by Wu et al. (2022), Wu X-L. et al. (2023) can be interpreted as a linear function of the logarithm of daily milk yield ( y ) with respect to milking interval time ( t ), DIM ( d ) where applicable, and the logarithm of a single milking yield ( x ), depicted as:

$$\log\left(y\right)=\alpha +\beta t+\gamma \left(d-d_{0}\right)+b\;\log\left(x\right)+\epsilon$$ (5)

Applying exponentiation on both sides of the above equation gives:

$$y=x^{b}e^{\left(\alpha +\beta t+\gamma \left(d-d_{0}\right)+\epsilon \right)}$$ (6)

Recognizing e≈2.718, we can re-arrange the above exponential function to the following, analogous to an exponential growth (or decay) function:

$$y=y_{0}{\left(1+r\right)}^{t^{*}}$$ (7)

where $y_{0}=x^{b}$ is the initial state, and r=1.718 designates the rate of change, modulated by a meta-time term $E\left(t^{*}\right)=\alpha +\beta t+\gamma \left(d-d_{0}\right)$ . Mathematically, y undergoes an exponential growth when $t^{*}>0$ , or an exponential decay when $t^{*}<0$ .

Wu et al. (2022) demonstrated that all these models gave similar daily yield estimates for milking intervals between 10 and 14 h. However, discrepancies in the estimated daily milk yields become pronounced outside this range. Specifically, ACF and MCF models were markedly superior to the baseline 2X model, producing drastically more minor errors and greater accuracies. The MCF models slightly surpassed the ACF models. Compared to the currently used methods, the exponential model stood out for its accuracy. For instance, relative to the Wiggans (1986) model, the exponential regression model boosted the precision of estimated daily milk yields by approximately 0.9% for Holstein cows and 1.5% for Jersey cows.

### Going beyond linearity

The Wiggans (1986) model, a *de facto* method for estimating daily milk yields in the United States, hinges on the assumption of linearity between proportional daily yields and milking interval time. Designed initially to determine MCFs for cows milked three times a day, the assumption stands due to the short milking intervals involved. However, Wu et al. (2023b) have recently demonstrated that this linearity assumption does not apply to cows milked twice daily, as longer, irregular milking intervals are involved. Historical data indicated that daily milk yield (including fat and solids-not-fat) did not have a linear relationship with milking intervals exceeding 12 h (e.g., Ragsdale et al., 1924; Bailey et al., 1955; Elliott and Brumby, 1955; Schmidt, 1960). Hence, early studies tended to postulate exponential milk curves up to 36 h (e.g., Brody, 1945). Klopcic et al. (2013) suggested a modified Michaelis-Menten function as a better fit for the trajectory of daily milk yields in relation to a milking interval over the same duration. Mathematically, the modified Michaelis-Menten function is an altered exponential function, where the base is one plus the yield for an interval of 720 min (i.e., 12 h), and the exponent is a non-linear function of milking interval time. Neal and Thornley (1983) showed that milk and component productions exhibited an exponential increase in relation to the interval between the current and the previous milkings, which later leveled off to an asymptote, potentially due to cell degradation and milk present in the udder.

Biologically, the milk secretion rate vitally influences the required frequency for milking cows and the acceptable intervals between milkings. The milk secretion rate depends on the pressure accumulating within the mammary gland. When milk builds up and accumulates in the mammary gland for a long enough time period, sufficient pressure is generated to inhibit secretion, leading to milk reabsorption by the blood (Schmidt, 1971). A marked increase in pressure occurs 1 hour after milking. Residual milk or complementary milk then moves from the alveoli into the teat and gland cisterns, causing a gradual increase in pressure due to the milk flow from the alveoli to the teat and gland cisterns. The rate of milk secretion remains linear for approximately 10–12 h after the last milking, then decreases slightly afterward. This decrease continues until it ceases around 35 h after the previous milking (Tucker et al., 1961; Schmidt, 1971). Therefore, while the linearity assumption is valid for approximately 12 h post-milking, it is not sustainable beyond.

Wu et al. (2023b) evaluated various non-linear modeling strategies in the Holstein dairy cattle. First, polynomial regression is a common non-linear model. Shook et al. (1980) used quadratic regression as a smoothing function to fit empirically computed MCFs in practical scenarios. Second, rather than fitting a single polynomial over the entire range of milking intervals, it is feasible to fit multiple polynomials over different segments of milking interval time. These segments are delineated by time points, known as “knots”, leading to piecewise polynomial regression. Positioning *k* knots throughout the milking interval range results in fitting *k*+1 distinct cubic polynomials. Introducing more knots defines a more flexible piecewise polynomial regression. With local smoothing, it is often necessary to enforce constraints for smoothness to ensure that the fitted curves are continuous and smooth at each knot. Mathematically, this implies that the first and second derivatives of the piecewise polynomial must be continuous at each knot, thus producing the third type of curve known as cubic splines. Splines can exhibit high variance at the extremes of the predictor range, primarily when milking interval times are very short or long. Hence, an additional constraint can be applied to mitigate this issue, compelling the function to be linear at the boundary. This method is known as natural splines. Likewise, step functions fit piecewise constant regression coefficients within different milk interval bins. We note that the model proposed by DeLorenzo and Wiggans (1986), denoted by (**D-W**), shares some commonalities with step functions, except that the D-W model does not define an intercept. Additionally, in the D-W model, the response variable is a test-day milk yield rather than a proportional test-day milk yield, and the predictor variable is a single milk yield (AM or PM yield) rather than the milking interval time. Lastly, Local regression, smoothing splines, and generalized additive models are three examples of promising models that can potentially improve the accuracy of estimated test-day milk yields (Wu et al., 2023b). Local regression, such as LOESS, allows for fitting flexible non-linear functions by computing a fit at a target point using only the nearby (“local”) observations (Cleveland, 1979). Local regression is also known as moving regression, a generalization of the moving average and polynomial regression (Harrell, 2015). Smoothing splines are functions that balance a measure of goodness of fit with a derivative-based measurement of smoothness (Craven and Wahba, 1979). In contrast, a spline tends to interpolate all the observed data points, which can lead to overfitting. Hence, a smoothing spline function can maintain the smoothness of the curve while minimizing the residual sum of squares. A GAM predicts the relationship between a response variable and one or more predictor variables while allowing for non-linear relationships. GAMs were initially developed by Hastie and Tibshirani (1990) to combine the properties of generalized linear models with additive models.

Mathematically, most non-linear models can generally be expressed using basis functions. For instance, consider *m* polynomials. The concept here is to have available a set of basis functions or transformations that can be applied to the variable of a milking interval t : $b_{1}\left(t\right)$ , $b_{2}\left(t\right)$ , . . ., $b_{1}\left(t\right)$ . That is,

$$z=\beta_{0}+\beta_{1}b_{1}\left(t\right)+\beta_{2}b_{2}\left(t\right)+\ldots +\beta_{m}b_{m}\left(t\right)+\epsilon$$ (8)

where $b_{j}\left(t\right)=t^{j}$ is the basis function for polynomial regression. Similarly, step functions accommodate piecewise constant regressions within different milk interval bins. Assume the milking interval is divided into K+1 bins, delimited by cutpoints $C_{1}$ , $C_{2}$ , …, and $C_{K}$ . Let $C_{k}$ represent a dummy variable for the *k*-the milking interval bin. Its value is one when the milking interval falls within the *k-*the milking interval bin and zero otherwise, for k=0,1,2,…,K . The step functions are defined as follows:

$$z=\beta_{0}+\beta_{1}C_{1}\left(t\right)+\beta_{2}C_{2}\left(t\right)+\ldots +\beta_{K}C_{K}\left(t\right)+\epsilon$$ (9)

Here, $C_{1}\left(t\right)=C_{2}\left(t\right)=\ldots =C_{K}\left(t\right)=0$ for the first milking interval bin and $C_{1}\left(t\right)+C_{2}\left(t\right)+\ldots +C_{K}\left(t\right)=1$ otherwise, meaning that the partial yield needs to be in only one of the following K milking intervals. In the above equation, $\beta_{0}$ is the mean value of $z_{i}$ for the first interval ( $t<c_{1}$ ). Then, for $t\geq c_{1}$ , Equation 10 becomes $z=\beta_{0}+\beta_{k}+\epsilon$ . Hence, the above equation predicts the response of $\beta_{0}+\beta_{k}$ for $c_{k}\leq t<c_{k+1}$ , in which $\beta_{k}$ represents the average change in the response for *T* in $c_{k}\leq t<c_{k+1}$ relative to $t<c_{1}$ . The basis functions for the piecewise constant regression model are the following:

$$b_{j}\left(t\right)=I\left(c_{j}\leq t<c_{j+1}\right)$$ (10)

Here, $I\left(c_{j}\leq t<c_{j+1}\right)$ is an indicator function, which equals one if the condition $c_{j}\leq t<c_{j+1}$ holds, and zero otherwise. Observing that the D-W model consists of local functions, we can similarly construct the basis function as follows:

$$z=b_{1}\left\{C_{1}\left(t\right)x\right\}+\beta_{2}\left\{C_{2}\left(t\right)x\right\}+\ldots +\beta_{k}\left\{C_{K}\left(t\right)x\right\}+\epsilon_{i}$$ (11)

where $C_{k}\left(t\right)x=x$ if $C_{k}\left(t\right)=1$ or $C_{k}\left(t\right)x=0$ if $C_{k}\left(t\right)=0$ , for k=1,2,…,K .

Finally, a cubic spline with *k* knots can be represented using a basis function. One possible form is a truncated power basis defined per knot, as follows:

$$h\left(t,c\right)={\left(t-c\right)}_{+}^{3}=\left\{\begin{array}{c} \begin{array}{cc} {\left(t-c\right)}^{3} & if\;t>c \end{array}\\ \begin{array}{cc} \;0 & \;otherwise \end{array} \end{array}\right.$$ (12)

where *c* is the knot. To fit a cubic spline with *K* knots to the data, we perform a least squares regression with an intercept and 3+K predictors: $t,t^{2},t^{3},h\left(t,c_{1}\right),\ldots ,h\left(t,c_{K}\right)$ . The above amounts to estimating K+4 regression coefficients.

Mathematically, GAMs do not represent a different form of non-linear functions, but they provide a generalized model framework that additively accommodates non-linear functions or predictors. Consider milking interval ( t ), DIM ( d ), and parities ( p ) as the predictor variables. Then, GAM for predicting daily milk yields can be set up as follows:

$$g\left(E\left(z\right)\right)=\beta_{0}+f_{1}\left(t\right)+f_{2}\left(d\right)+f_{3}\left(p\right)$$ (13)

In the above, $E\left(z\right)$ is the expected value of the response variable, *g* is a link function that relates the expected value of the response to the linear predictor, $\beta_{0}$ is the intercept term, and $f_{1}\left(t_{i}\right)$ , $f_{2}\left(d_{i}\right)$ , and $f_{3}\left(p_{i}\right)$ are the smooth functions of the three predictor variables, respectively. Here, t and d are treated as continuous variables, and p is a categorical variable. Note that milking interval time can be discretized and treated as a categorical variable. The link function, g , is usually an exponential distribution, such as a normal (i.e., Gaussian), binomial, or Poisson distribution. Conveniently, an identity function can be the link function such that $g\left(E\left(z\right)\right)$ reduces to $E\left(z\right)$ .

**FIGURE 2:**
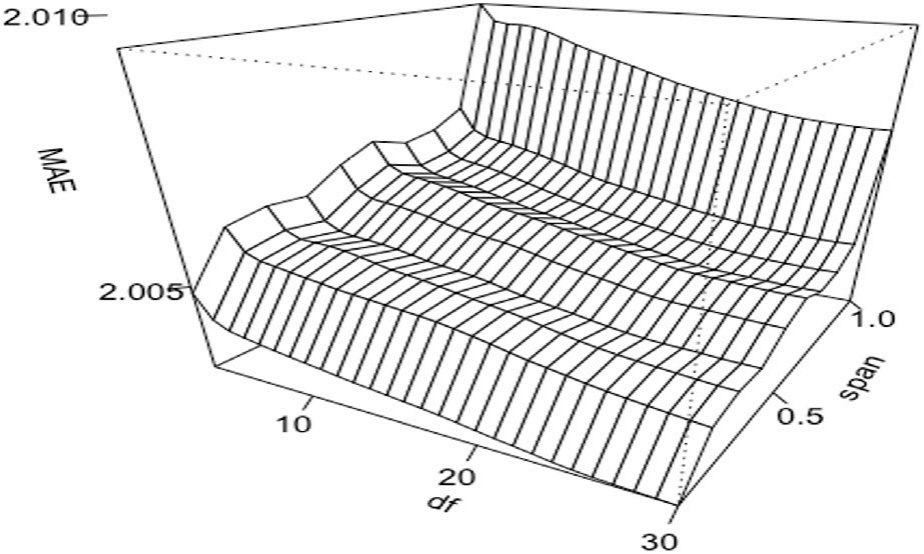
Three-dimensional relationships contour plots of mean absolute errors (MAE) of estimated daily milk yields obtained from a generalized additive model that fitted proportional test-day milk yields ( z ) on milking interval time ( t ) as locally weighted regression ( loess ) with varying span parameter values (from 0.1 to 1.0) and DIM ( d ) as cubic splines ( cs ) varying degrees of freedom ( df = 3:30).

Non-linear model performance often rests on the appropriate selection of hyperparameter values. Figure 2 shows the effects of two distinct hyperparameters with GAM. In this example, we assigned LOESS to fit the milking interval with the span value varying from 0.1 to 1.0 and employed smoothing splines for DIM, modulating its degrees of freedom between 3 and 30. The smoothing splines are, in essence, natural cubic splines with knots set at every distinct predictor variable value—the milking interval time. The degrees of freedom in these splines influence the penalized likelihood’s shrinkage and the splines’ overall smoothness. For LOESS, the span parameter determines the data percentage used in the local regression, with a smaller value featuring a localized regression and a larger value catering to a more global regression. The results showed that the mean absolute errors (**MAE**) were smaller with a small span value than a large value when fitting LOESS on milking interval time. With smoothing splines fitted on DIM, MAE was smaller with large degrees of freedom than with small degrees of freedom. Therefore, the most optimal values of these two parameters are span = 0.1 for LOESS and 30 degrees of freedom for the smoothing splines in this example.

Wu et al. (2023b) compared the performance of the aforementioned non-linear models for estimating daily milk yields. Overall, these non-linear models, except step functions, demonstrated smaller errors and greater accuracies for estimated test-day milk yields to varying extents compared to the traditional linear models. Among the non-linear models, GAMs yielded the least bias and the greatest accuracy. This accentuates the potential of further harnessing GAMs to estimate test-day milk yields. Other non-linear models which are also promising include LOWESS and smoothing splines.

## Deriving daily yield correction factors

### Discrete additive factors

Additive correction factors are calculated for discrete milking interval classes, with the additive correction amount quantified by ∆=α+βt . For instance, the ACF for the *k*th milking interval class is computed as follows:

$${∆}^{\left(k\right)}=\hat{\alpha }+\hat{\beta }{\bar{t}}^{\left(k\right)}$$ (14)

where ${\bar{t}}^{\left(k\right)}$ is a midpoint of milking interval class *k*. Here, we used the superscript “(k)” as an index for discretized milking interval classes.

Wu et al. (2022), Wu X-L. et al. (2023) show that the sum of ACFs within each milking interval class was constant. For traditional AM-PM ACF models, where the regression coefficient for the partial yield is b=2 , the sum is zero:

$$\Delta_{AM}^{\left(k\right)}+\Delta_{PM}^{\left(k\right)}=0$$ (15)

For linear regression, where b can take real values, the sum is the following.

$$\Delta_{AM}^{\left(k\right)}+\Delta_{PM}^{\left(k\right)}=\left(2-b\right){\bar{y}}^{\left(k\right)}$$ (16)

Using a simulation dataset, Wu X-L. et al. (2023) showed that the sum of ACFs within each milking interval class was 0 for conventional ACF models ( b=2 ), and it was 1.405 for the linear regression model ( b=1.942 ). This larger sum with linear regression arose because its regression coefficient for the partial yield in linear regression was less than 2. In this example, the traditional ACF model supplemented additive corrections to 2 times the partial yields, whereas the linear regression supplemented additive corrections to 1.942 times the partial yield. Knowing their relationships provides convenience for calculating daily yield correction factors. For example, once evening ACFs are calculated, then morning ACFs can be conveniently obtained using the relationships (15) and (16), and *vice versa* (Shook et al., 1980).

### Discrete multiplicative factors

Multiplicative correction factors are also calculated for discrete milking intervals. For the DeLorenzo and Wiggans (1986) model, the regression coefficient is obtained for each milking interval class, denoted by $b^{\left(k\right)}$ , which coincides with the corresponding MCF. An alternative form of MCF based on this model is as follows:

$$F^{\left(k\right)}=b^{\left(k\right)}=\frac{E\left(y^{\left(k\right)}\right)}{E\left(x^{\left(k\right)}\right)}=\frac{\frac{1}{n}\sum y^{\left(k\right)}}{\frac{1}{n}\sum x^{\left(k\right)}}=\frac{\sum y^{\left(k\right)}}{\sum x^{\left(k\right)}}$$ (17)

The above form of MCFs aligns with that empirically derived by Shook et al. (1980). Both share the same interpretation of an MCF as the ratio of the expected value of daily milk yields over the expected value of a single (AM or PM) milk yield. Shook et al. (1980) evaluated MCFs empirically as the ratio of bulk daily milk yield to bulk AM or PM milk yield. In the above, we observe that $\sum x^{\left(k\right)}$ corresponds to bulk AM or PM milk yields and $\sum y^{\left(k\right)}$ corresponds to daily milk yields.

For the Wiggans (1986) model, MCFs are obtained by locally taking the expected value on both sides of the model equation, assuming that $E\left(\epsilon \right)=0$ . That is,

$$F^{\left(k\right)}=E\left(\frac{y^{\left(k\right)}}{x^{\left(k\right)}}\right)=\frac{1}{\hat{\alpha }+\hat{\beta }{\bar{t}}^{\left(k\right)}}$$ (18)

The above MCF corresponds to the expected value of the ratio of a test-day yield to a partial yield, not the same as that derived from DeLorenzo and Wiggans (1986). Still, both forms coincide with each other approximately because taking the first-order Taylor series approximation to the term on $E\left(\frac{y^{\left(k\right)}}{x^{\left(k\right)}}\right)$ leads to an approximation: $E\left(\frac{y^{\left(k\right)}}{x^{\left(k\right)}}\right)\approx \frac{E\left(y^{\left(k\right)}\right)}{E\left(x^{\left(k\right)}\right)}$ .

The relationship between morning and evening MCFs, confined to the same dataset, is the following (Wu X-L. et al., 2023):

$$\frac{1}{F_{AM}^{\left(k\right)}}+\frac{1}{F_{PM}^{\left(k\right)}}=1$$ (19)

Multiplicative correction factors for non-linear models, such as the exponential regression model, can also be constructed, e.g., by following the second interpretation of DeLorenzo and Wiggans (1986). Please refer to Wu et al. (2023a) for a detailed description.

### Continuous correction factors

The practice of deriving daily yield correction factors on discrete milking interval classes has been in place for decades, but it was recently under scrutiny (Wu et al., 2023b). On the one hand, calculating correction factors on discrete milking interval bins presumes that these factors remain consistent within each bin, imposing a theoretically debatable contention. On the other hand, determining the optimal size for these discrete bins is practically challenging. A bin size that is too small might not provide enough data to calculate yield correction factors for every bin accurately. Conversely, a large bin size can inevitably compromise the accuracy of the estimated daily milk yields.

Instead, continuous daily yield correction factors can be derived (Wu et al., 2023b). For example, assuming there is sufficient data for every milking interval time range, continuous MCFs can be derived based on the Wiggans (1986) model as follows:

$$F_{t^{*}}=\frac{1}{E\left(\left.z\right|{\hat{\beta }}_{0},{\hat{\beta }}_{1},t=t^{*}\right)}=\frac{1}{{\hat{\beta }}_{0}+{\hat{\beta }}_{1}t^{*}}$$ (20)

By noting ${\hat{z}}_{t^{*}}={\hat{\beta }}_{0}+{\hat{\beta }}_{1}t^{*}$ , where z is a proportional daily milk yield, we can calculate an MCF as the reciprocal of average estimated proportional daily yields, utilizing all data satisfying $t=t^{*}$ . That is,

$$F_{t^{*}}=\frac{1}{E\left(z|t=t^{*},\hat{\theta }\right)}=\frac{1}{{\hat{z}}_{t^{*}}}$$ (21)

Here, θ collectively includes all unknown model parameters. The above formula retains the MCF interpretation by the Wiggans (1986) model, assuming all other covariates have been averaged out or are non-existent. Because $E\left(z|t=t^{*},\hat{\theta }\right)$ can be evaluated numerically by estimated or fitted proportional daily yields, Equation 21 is universally applicable, encompassing both linear and non-linear models, and even non-parametric models.

In practice, however, there may not always be sufficient data for every milking interval time unit. A more favorable approach, in line with the concept of locally weighted regression, involves calculating the MCF for each milking interval time t by utilizing the data within locally defined neighborhoods, not limited to using only the data with $t=t^{*}$ (Wu et al., 2023b). Then, the MCF can be obtained by taking the reciprocal of the weighted mean of proportional daily milk yields within each locally defined neighborhood:

$$F_{t^{*}}=\frac{1}{E\left(f\left(w^{\prime }z|\theta ,t\in N\left(t^{*}\right)\right)\right)}$$ (22)

where $t\in N\left(t^{*}\right)$ comprises all the observations within the neighborhood centering at $t^{*}$ , and w is a vector of weights defined for all the observations in this neighborhood. The weights are defined to be linearly or non-linearly proportional to a distance measure in milking interval time for each observation relative to $t^{*}$ . Think of deriving continuous MCFs on moving windows, which are neighborhoods. The size of the neighborhood is adjustable, depending on the data information required. Within each neighborhood, the weight increases as the observation gets close to $t^{*}$ in milking interval time. Hence, with the local regression approach, computing continuous MCFs can overcome the limitations of the discrete MCF model because it can always ensure sufficient data for computing MCFs and minimize system bias.

**FIGURE 3:**
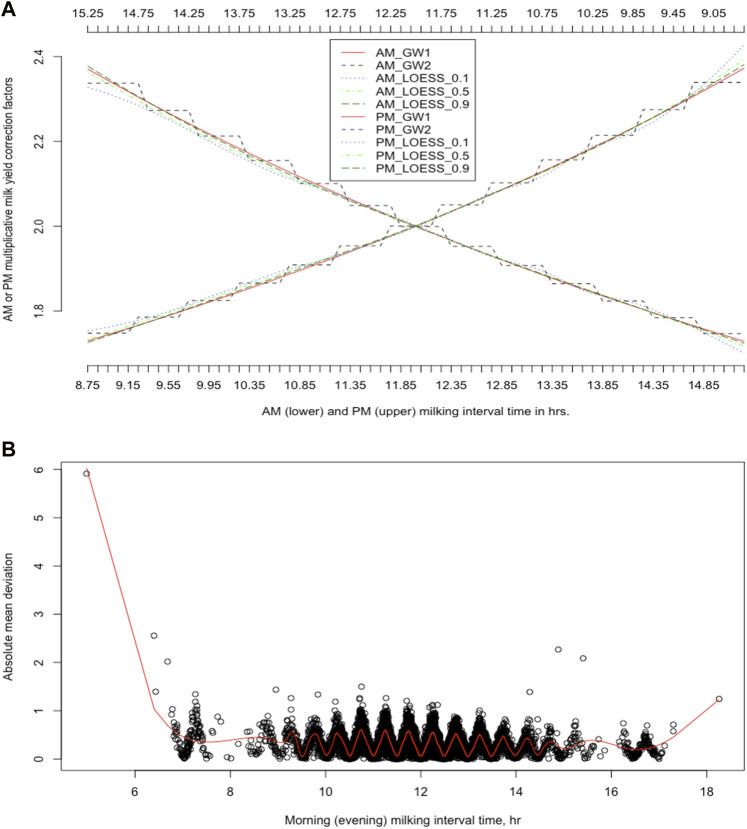
Multiplicative milk yield correction factors (MCFs) obtained using two different strategies **(A)** Discrete *versus* continues MCFs for the morning (AM) and evening (PM) milkings, derived from the Wiggans (1986) model (GW) and locally weighted regression (LOESS; span = 0.1, 0.5, and 0.9, and degree = 1) **(B)** Discrepancies between smoothed average test-day milk yields and average estimated test-day milk yields obtained based on MCFs obtained using the Wiggans (1986) model. GW1 = MCFs computed for every 0.01-h time unit based on the Wiggans (1986) linear regression model; GW2 = MCFs computed for 30-min bins based on the Wiggans (1986) model; LOESS_ α = MCFs calculated for every 0.01-h time unit based on LOESS with the span being α = 0.1, 0.5, 0.9, respectively.

Figure 3A shows MCFs calculated with both approaches using a simulation dataset (Wu X-L. et al., 2023). Continuous MCFs were generated every 0.1-h time using LOESS and the Wiggans (1986) model (**GW1**). The Wiggans (1986) model was also used to generate discrete MCFs for 30-min milking interval bins (**GW2**). Overall, the MCFs obtained from GW1 were more akin to the MCFs obtained from LOESS with larger span parameter values (0.9) because LOESS with large span parameter values featured global regression. Nevertheless, both models did not give identical MCFs, suggesting that they still performed differently. The weights assigned to each dataset with LOESS are different: the closer a data point was to the mid-point of the neighborhood, the larger weight it got. In contrast, GW1 assigned the same weights to all data points. Therefore, LOESS can achieve the best local fitting while retaining excellent global smoothing.

To show the presence of systematic biases in daily milk yields estimated from discrete MCFs, we calculated average estimated daily milk yields thus obtained with the Wiggans (1986) model and, meanwhile, fitted smoothing splines on the actual test-day milk yields and calculated average estimated test-day milk yields. The absolute deviation between the smoothed average of actual daily milking yields and the average of estimated daily milk yields was minimal at the mid-point of each milking interval class or bin; it then increased as the milking interval time moved away toward the boundaries (Figure 3B). Similar results were also obtained using the real dataset in the US Holstein dairy cattle (Wu et al., 2022). These recurrent patterns suggest that systematic biases were minimal at the central location of each milking interval bin and maximum on the boundaries between milking interval bins. Wu et al. (2022), Wu et al. (2023a), Wu et al. (2023b) have analytically shown why large milking interval classes or bins can lead to systematic errors. All these results, when combined, forcefully suggest that the periodical biases arising from deriving correction factors on large discrete milking interval classes were not just theoretical. Still, it is playing out in the real world, too.

## Applications to genetic evaluations

Utilizations of test-day milk yields in genetic evaluations fall into two broad categories. In countries such as Canada, Finland, Germany, Italy, the Netherlands, and Switzerland, genetic evaluations are performed directly based on test-day yield records. This approach is referred to as the test-day yield model or, simply, the test-day model. In contrast, the United States employs a distinct strategy wherein genetic evaluations are derived from projected 305-day lactation yields, calculated from a subset of test-day yields throughout the lactation period. The latter method is referred to as the lactation yield model. Notably, test-day data have been collected in the United States since 1905 for management and have contributed to national genetic evaluations since 1936. However, the patenting of the test-day model in the United States in 1993 confined its application primarily to regional and unofficial evaluations under the exclusive purview of the patent holders (Powell and Norman, 2006).

### Lactation yield model

A lactation yield model capitalizes on the high heritability of total lactation yield, providing an assessment directly pertinent to dairy producers. The total amount of milk produced over a lactation is a direct determinant of farm revenue. However, an intrinsic drawback of a lactation model is that it does not utilize all available test-day records, though lactation curves can be inferred by additional steps based on test-day milk yields. Knowing individual lactation curves can be useful in making informed breeding decisions.

***Projection of lactation milk yields*** Accurately projecting total lactation milk yields is crucial to genetic evaluations under the lactation milk yield model. The Centering Data Method (**CDM**; McKellip and Seath, 1941) and the Test Interval Method (**TIM**; Sargent et al., 1968) were two well-known empirical methods used to estimate lactation milk yields in the 20th century. CDM calculated lactation yields based on yields from two consecutive milkings per month. The sampling day was centered as nearly as possible in the test month period but not necessarily aligned with the calendar month. Nevertheless, CDM overestimated actual yields until peak lactation and the yield following the last test day, typically underestimating yields for other test periods. Consequently, TIM supplanted CDM in the US in 1969 (McDaniel, 1969). TIM interprets the span between two test days as a distinct test period, with production credits bifurcated based on the data from each test day. Production credits for the first half of the test period are computed based on the first test-day information. Production credits for the second half of the test period were based on the second test-day information. For the first and last test intervals, yield credits were calculated similarly to CDM. Hence, TIM produced more accurate estimates when milk weights and component samples were obtained monthly, permitting greater flexibility than CDM (Norman et al., 1999). Shook et al. (1980) further proposed adjusting the credits for the first and second test intervals for the nonlinear shape of the lactation curve and the last test for a continuation of the expected decline, aiming to minimize the biases from overestimating credits for the first and last test intervals and underestimating credits for the second test interval.

Beginning in February 1999, the Best Prediction (**BP**) methodology was employed to estimate unobserved daily yields based on known daily yields (VanRaden, 1997; Cole and VanRaden, 2006). A fundamental assumption underlying BP is the prior knowledge of means and (co)variances, coupled with the premise that deviations of observed and unobserved milk yields from their respective population means follow a multivariate normal distribution. Consequently, the 305-day milk yield is calculated by the sum of the population mean and the product of covariance between observed and unobserved test-day yields, the inverse variance of observed test-day yields, and the deviations of observed test-day yields. The population means are often derived from the population average of lactation curves, for instance, the Wood lactation curve (Wood, 1967). Studies have shown that BP yielded more accurate 305days yields than TIM (e.g., Norman et al., 1999). Aggregating all the measured and estimated daily yields up to 305 days in milk gives the lactation yield. An added advantage of this aggregation method is its capacity to facilitate inference on individual lactation curves. Alternatively, lactation milk yields can be calculated directly without aggregating daily yields. BP was initially implemented limited to 305-day lactations. However, longer lactations can be accommodated by estimating covariances for days in milk greater than 365 days (Cole et al., 2007; Cole et al., 2009). Lactation milk yields can be estimated via a single-trait prediction exclusively from test-day milk yields or through multi-trait analysis encompassing other component traits, such as fat and protein. Multiple-trait predictions exhibit heightened accuracy, especially when some component samples are lacking (Schaeffer and Jamrozik, 1996).

*Standardization of lactation records* In the United States, lactation records have been standardized to a mature equivalent yield since the beginning of the 20th century (Freeman, 1971). Adjustments to age metrics other than mature age have also been considered (e.g., McDaniel, 1973). A mature-equivalent lactation yield delineates the hypothetical amount of milk a cow would have produced if she were mature (roughly 4–5 years old) under a twice-daily milking regime for 305 days. Milk records extending beyond 305 days are truncated, whereas those falling short are projected to 305-day yields (VanRaden, 1997). The key to adjusting milking frequencies is quantifying the relative increase of daily yields when switching from non-standard milking to standard, twice-daily milking. Notably, such gains can vary with age, season, and region. Wiggans and Powell (1980) estimated the relative increase in milk yield from 3X milking compared to 2X milking daily, which was 20% for 2-year-old cows, 17% for 3-year-old cows, and 15% for 4-year-old cows. Furthermore, a recent proposal by VanRaden et al. (2023) advocates a negative exponential function to derive correction factors tailored for adjusting milking frequencies. It is evident that current correction factors for adjusting milking frequencies are outdated and necessitate updating. Powell and Norman (2006) also argued against adjusting milking frequencies independent of multiplicative independent of milk yield standardization for other factors, positing that discerning yield variations solely based on milking frequency is challenging.

Additionally, lactation standardization considers factors such as age of the cow (or age-parity), and month of the year at calving (or season-region) (Norman et al., 1995; Miles et al., 2023). The mature-equivalent factors currently used in the national evaluation were derived almost 30 years ago (Schutz and Norman, 1994). The USDA updated age factors routinely in 1935, 1942, 1953, and 1974. Norman et al. (1995) reported changes in maturity patterns across time. Adjustments catering to 5-year periods were integrated into the national animal model from 1995 onwards. Multiplicative factors standardize the mean and standard deviation proportionally, and separate pre-adjustment factors standardize the genetic variances of records across time and herds before their incorporation into the animal model (Wiggans and VanRaden, 1989). A further pre-adjustment was added in 2007 to standardize variance across parities. Notably, the base adjustment varies with countries. For instance, while the United States, Australia, Canada, and Italy adjust records to mature age, some other countries opt for adjusting records to a first-lactation age ranging from 24 to 30 months. Israel adjusts to 36 months or the age of average production. Recommendations from Interbull lean towards adjusting to the average age. While the rationale for mature age adjustments may be entrenched in tradition, adjustment to average age may be more realistic because it puts records on the scale of an average cow in the herds, and, on average, adjusted and actual yields would be similar for a herd (Powell and Norman, 2006). Nevertheless, as Miller (1973) analytically highlighted, for any set of age means from which adjustment factors are constructed, the base to which records are adjusted exerts minimal influence on ranking animals.

*US dairy genetic evaluations* In the United States, the genetic merits of animals are assessed on mature equivalent 305-day lactation yields, utilizing a multiple-trait, animal model (Wiggans et al., 1988; Wiggans and VanRaden, 1989; VanRaden et al., 1995). Incoming data undergo rigorous checks for plausible values and alignment with pre-existing records (Norman et al., 1994; Miles et al., 2023). Multiplicative adjustments are conducted for calving age and month within each breed, times milked per day (adjusted to twice daily milking), previous days open, and heterogeneous variance. The base for mean and variance adjustments is set at 36-month-old and second-parity cows. Unequal variances across time, herds, and breeds are adjusted to the base variance calculated from standardized records of first lactation cows that calved in 2007.

The current CDCB genetic evaluations are scheduled tri-annually (April, August, December), encompassing lactation records from animals that calved post-1960, with the pedigree data from birth years as far back as 1950. The animal model, operational since 1989, is represented as:

$$y=Mm+Za+ZA_{g}g+Pp+Cc+e$$ (23)

In this model equation, y is a vector of standardized lactation records (e.g., milk, fat, and protein yields). Vectors ***m***, a , g , p , and c contain the effects for the management group, random additive genetic merit, unknown parent group, permanent environment, and herd-sire, respectively. Matrices M , Z , $ZA_{g}$ , P **,** and C denote their respective incidence matrices, while e encapsulate unaccounted residuals. This model facilitates the simultaneous evaluation of all animals in the dataset, accounting for all relatives that contribute to the assessment of each animal (VanRaden and Wiggans, 1991). Unknown parent groups can also be considered (VanRaden and Wiggans, 1991), The genetic evaluation system has been modified at times to meet the needs of the US dairy industry and leverage technical and methodological advancements. The current animal model has been enhanced to capture phenotypic variations due to varying calving ages and parities. Unknown parents are grouped by birth years, breeds, and, for Holstein cattle, separately for US and foreign animals. Unknown sires and dams of cows are grouped separately, but unknown parents of bulls are assigned to a combined group. The relationship matrix also accounts for the effects of inbreeding on Mendelian sampling variance. The genetic base in the US dairy cattle evaluations is recalibrated every 5 years. Hence, the next base change will be in 2025, when the cows born in 2020 become the base population, and their average evaluation will be set to 0. The periodic base recalibration, executed every 5 years, is integral for reflecting the genetic progress observed in the population.

### Test-day yield models

A test-day model is an animal model directly evaluating test-day observations. These models accommodate diverse data structures, from varied intervals to distinct recording plans. For instance, while some herds may only record milk yields, others may also include records of fat and protein contents. Test-day models offer a nuanced way to account for factors with varying effects on each test day (Druet et al., 2003). Moreover, specialized curves for distinct factors can be deduced by nesting the DIM class into the source of variation (Stanton et al., 1992). Historically, two-step test-day models emerged in regions such as Australia (Beard, 1983; Jones and Goddard, 1990), New Zealand (Johnson, 1996), and the Northeastern United States (Kachman and Everett, 1989; Everett et al., 1994; Wiggans and Gengler, 1999). This methodology first corrects test-day records for factors such as age-season, previous open days, milking regularity, lactation stage, milking age, and gestation days. Post-adjustments, breeding values for lactation traits are then ascertained via an animal model. On the other hand, one-step models perform both processes concurrently. The latter approaches fall into two categories. The first category includes test-day models with fixed regression of yield on DIM, assuming that test-day records within a lactation are repeated records (Meyer et al., 1987; Ptak and Shaeffer, 1993), hence referred to as repeatability test-day (**REP-TD**) models. The second category includes random regression test-day (**RR-TD**) models that define the animal’s genetic effect by random regression coefficients, also allowing for covariances among them.

*Repeatability test-day models.* Meyer et al. (1987) originally proposed one-step REP-TD models in Australia in the form of a sire model. Ptak and Schaeffer (1993) advocated using a repeatability animal model for genetic evaluations of dairy sires and cows, making it popular. A general scalar representation of a REP-TDM is the following:

$$y=HTD+\sum b_{i}x_{i}+a+p+e$$ (24)

where *y* is a test-day yield, *HTD* is a fixed herd test-day effect, *a* is a random genetic effect of an animal, *p* denotes the permanent environmental effect associated with each lactation, and *e* is the residual term. The lactation curve is accounted for by several coefficients of a fixed regression of yield on DIM or functions of DIM, $\sum b_{i}x_{i}$ . Optionally, regressions can be nested within classes of other fixed effects like age. This ensures that the heterogeneity of the residual variance is accounted for (Ptak and Schaeffer, 1993). This model has been extended to multiple traits such as milk, fat, protein, and somatic cell scores, with test-day records within the lactation viewed as repeated traits and those between lactations as separate traits (Reents et al., 1995a; Reents et al., 1995b).

*Random regression test-day models.* A REP-TD model is ideal if genetic correlations among test-day yields are very high or near unity. But, in reality, they are not. To better fit the latter scenario, Schaeffer and Dekkers (1994) proposed using random regressions for evaluating animals’ genetic effects, permitting a covariance structure among the regression coefficients. Their model underwent further refinements, culminating in Canada’s adoption of a multiple-trait (milk, fat, protein, and somatic cell score), random regression test-day animal model in February 1999 (Jamrozik et al., 1997b; Schaeffer et al., 2000). Typically, the model includes fixed regressions accounting for similarities of lactation curves within specified groups of animals (e.g., regions and age classes), and random regressions are added to account for the individual variation of animal genetic effects and permanent environmental effects (Jamrozik et al., 1997a; Jamrozik and Schaeffer, 1997). A similar model was proposed by Kettunen et al. (1998), which included a random herd test month of production effect with different submodels for the genetic and permanent environmental components. Gengler et al. (1999) proposed an RR-TD model with an alternate strategy for solving the system of equations. The general concept of random regressions was previously described by Henderson (1982) and Laird and Ware (1982).

The general scalar representation of the RR-TD model adopted in Canada (Jamrozik et al., 1997c; Schaeffer et al., 2000) is the following:

y=HTD+∑bz+∑az+∑pz+e (25)

where *y* is a test-day record, *HTD* is the fixed herd test-day effect, ***b*** is a vector of fixed regressions within region, age, and season, ***a*** is a vector of random regression genetic coefficients specific for each animal, ***p*** is a vector of random regression coefficients for permanent environmental effects for each cow, and *e* is the residual term for each observation. The submodel for the shape of the lactation curve is identical for fixed and random regressions. For instance, when Wilmink’s function is used (Wilmink, 1987), the function is defined by $z^{\prime }=\left(\begin{array}{ccc} 1 & t & e^{-0.05t} \end{array}\right)$ with *t* denoting DIM. Extending the RR model to simultaneously account for multiple traits (e.g., milk, fat, protein, and SCS) is straightforward but can be computationally intensive.

*Estimated breeding values and persistency proofs.* The REP-TD model operates on the premise that genetic variation remains constant throughout lactation. As a result, while EBVs can be computed for any stage of lactation, their interrelationships result in a basic linear function when calculated for distinct periods. On the other hand, RR-TD models permit changes in an individual’s genetic merit at any given day during lactation. Hence, an RR-TD model calculated the breeding value for an animal as integrals from the individual curve, enabling EBVs to be presented as curves of genetic merit (White et al., 1999). The inherent advantage of genetic merit curves lies in their capacity to visually portray genetic merit level while simultaneously depicting lactation persistency of lactation (Swalve, 2000). This feature aids breeders in selecting bulls most suitable for their production systems, especially when contemplating the optimal lactation length, even if it falls short of the standard 305-day lactation duration.

Persistency proofs can be deducted from the daily genetic merit curves derived from RR-TD models (Jamrozik et al., 1997b; Jamrozik et al., 1998). Persistency, an economically pivotal trait, influences feed expenses, health, and fertility traits (Dekkers et al., 1998). Of these aspects, the repercussions of persistency on health, specifically metabolic stress causing health problems in cows, may outweigh its effect on feeding costs. Assessing feed expenses involves determining how supplementing concentrates to persistent cows can be partially offset by roughage, thus reducing overall costs (Swalve and Gengler, 1998).

Random-regression test-day models allow for estimating the genetic variance and ‘genetic yields’ for each individual day of lactation, thus paving the way for establishing precise persistency benchmarks. Such adaptability allows diverse persistency criteria to be determined from genetic assessments with RR-TD models. For instance, Jamrozik et al. (1998) proposed using the average slope of an animal’s lactation curve between days 60 and 280 as a measure of persistency. Their findings pointed to heritability levels in the range of 0.20–0.30 for milk, fat, and protein yields over the first three lactation cycles, alongside an almost negligible genetic interrelation between persistency and yield. However, a challenge surfaced from their analysis: the genetic correlations of persistency across lactations remained consistently low, roughly around 0.35. The underpinnings for this tenuous interplay between lactations remain speculative. Indirect selection predicated on such feeble correlations would prove inefficacious. Further complexities emerge, such as the challenge of achieving consensus on a singular persistency definition, like the slope of the lactation trajectory between days 60 and 280, which opposes the aspiration of providing EBV for diverse production systems.

### Cross-country genetic evaluations

During the 1980s, exporting North American semen to multiple countries led to numerous globally located daughters of highly ranked bulls. This widespread distribution fueled the interest in evaluating bull merits internationally. In 1994, the International Bull Evaluation Service (Interbull), rooted in Sweden, was founded by four Nordic countries and incorporated two breeds.

Interbull harmonized national genetic evaluations from various countries to present assessments of a comprehensive set of bulls based on each participating country’s metrics. In August 1995, Interbull adopted the multiple-trait, cross-country evaluation system (MACE) proposed by L. R. Schaeffer (1994), which encompasses genetic correlations between countries that are less than unity. This model fundamentally functions as a single-trait sire (lactation) model, integrated with a vector of phantom parent genetic group effects, and is designed to facilitate the comparison of dairy sires across multiple nations.

$$y_{i}=\mu_{i}1+Z_{i}QG_{I}+Z_{i}S_{I}+e_{i}$$ (26)

In the above, $y_{i}$ is a vector of sire daughter yield deviations (**DYD**) from country *i*, $\mu_{i}$ is the overall average DYD, which reflects the definition of genetic basis in that country, $g_{i}$ is a vector of phantom parent genetic group effects, defined across countries and by birth year within the country of birth, $s_{i}$ is a vector of sire genetic effects (transmitting abilities), $Z_{i}$ is the matrix that relates elements of $y_{i}$ to elements in $s_{i}$ , Q is a matrix that associates sires with their genetic groups, and $e_{i}$ is a random mean residual effect defined for a variable number of daughters.

Subsequent model advancements encompass a multiple-trait, sire (lactation) model devoid of the phantom genetic group effect, as proposed by Weigel et al. (2001), and a multiple-trait, test-day (animal) model, as introduced by Jamrozik et al. (2002). In the former model, the fixed effects additionally include herd-year-season of calving, age at calving, milking frequency, and heterosis (breed composition) classes. In the latter model, the fixed effects integrate herd-test day effects and a combination factor compromising of breed composition, age at calving, season of calving, and DIM effect; the random effects include random regression coefficients for the permanent environmental effect, random regression coefficients for animal genetic effect, and regression coefficients for genetic group effects, in addition to the residuals.

## Aftermath: discerning actualities from projections

Estimated test-day milk yields have been used as if they were accurate depictions of actual test-day milk yields, neglecting the fact of possible estimation errors. The potential consequences arising from the disturbances linked to these estimates on subsequent genetic evaluations have not been sufficiently addressed. Liu et al. (2000) assessed six linear and non-linear regressions compared to the 2X method for estimating daily yields in AM-PM milking schedules. They reported a reduction in the variances of estimated yields compared to actual daily yields from different lactation stages, underscoring the need to expand the variance of estimated yields to a comparable scale with actual yields in genetic evaluation. Adjustments enabling comparable variances between actual and projected lactation yields were previously proposed by VanRaden et al. (1991). Essentially, linear (and quadratic) regression models are ACF models (Wu et al., 2022; Wu et al., 2023 X-L.). Hence, they did not preserve the actual variance structures as did MCF models (Miller, 1973). Even when estimated daily yields from single milkings were expanded to a comparable scale to actual daily yields, they could be assigned to more significant error variances than actual daily yields. Hence, variance-rescaling approaches could lower heritability and repeatability for expanded daily yields. Consequently, for estimating a cow’s breeding value, her own records received less weight when she was in AM-PM milking schemes than in a standard A4 testing program (Liu et al., 2000).

In this section, we analytically show the influence of errors associated with estimated test-day milk yields from two perspectives: estimating lactation milk yields using best prediction and genetic evaluation *per se*. In the former scenario, let y be a n×1 vector of 305-day milk yields for n animals, and X be a n×k matrix of test-day milk yield deviations, where *k* represents the number of test days for each lactation period. According to VanRaden (1997), the unknown lactation or daily yields are estimated to be a population average plus the covariance between 305-day milk yield and test-day milk yields ( c ) multiplied by the inverse of the variance of test-day yields ( $V^{-1}$ ), multiplied again by the test-day yield deviations. That is,

$$E\left(y\right)=1\mu +{\left(c^{\prime }V^{-1}X\right)}^{\prime }$$ (27)

where μ , c and V are assumed to be known. The above equation can be viewed as a linear regression yet with a known population mean and variance-covariance terms, which can be re-arranged as follows:

$$E\left(y\right)=1\mu +X\beta$$ (28)

where $\beta =c^{\prime }V^{-1}$ . The assumption for no measurement errors, typically made for linear regression, does not hold with (28) when applied to estimate 305-day yields. Test-day milk yields are not measured directly but instead estimated, introducing potential errors. When these estimation errors are small in magnitude, they can be safely discarded because their impact on the results tends to be minimal. However, if the errors are significant, they will lead to erroneous and even invalid estimates.

Denote projected lactation yields by $y^{*}$ , estimated from test-day yields, denoted by $X^{*}$ . Further, let test-day milk yields be estimated from single milkings with errors. Hence, errors-in-variance models (Griliches and Ringstad, 1970; Chesher, 1991) can be used to describe their relationships, as follows:

$$y^{*}=E\left(y\right)+\epsilon =1\mu +X\beta +\epsilon$$ (29)

$$X^{*}=X+V$$ (30)

Here, ϵ is a n×1 vector containing the usual disturbance, and V is a n×k matrix with measurement errors. We assume that ϵ and V both have null means and are mutually independent: $E\left(\epsilon \right)=0$ , $E\left(\epsilon^{\prime }\epsilon \right)=I\sigma_{\epsilon }^{2}$ , $E\left(V\right)=0$ , $E\left(V^{\prime }V\right)=R$ , and $E\left(V^{\prime }\epsilon \right)=0$ .

Replacing X in (29) with $X=X^{*}-V$ leads to:

$$y=1\mu +\left(X^{*}-V\right)\beta +\epsilon =1\mu +X^{*}\beta +\left(\epsilon -V\beta \right)=1\mu +X^{*}\beta +\epsilon^{*}$$ (31)

where $\epsilon^{*}=\left(\epsilon -V\beta \right)$ is a new error term, also referred to as the composite disturbance. The problem with (31) is that, assuming $E\left(\epsilon^{*}\right)=0$ , it infringes upon the fundamental assumption of linear regression because the explanatory variables and the error term are no longer uncorrelated, as follows:

$$E\left[{\left(X^{*}-E\left(X^{*}\right)\right)}^{\prime }\left(\epsilon^{*}-E\left(\epsilon^{*}\right)\right)\right]=E\left[V^{\prime }\left(\epsilon -V\beta \right)\right]=E\left[V^{\prime }\epsilon \right]-E\left[V^{\prime }V\right]\beta =0-R\beta =-R\beta \neq 0$$ (32)

When using the estimated test-day milk yields with measurement errors to estimate the actual lactation yields, the resulting linear regression coefficients also do not correspond to their “true” values. For instance, let $x_{j}^{*}=x_{j}+v_{j}$ , where $x_{j}^{*}$ and $x_{j}$ are vectors for estimated and actual milk yields on test day *j*, respective, and $v_{j}$ is a vector of estimation errors specific to the *jth* test day. Let $y=1\mu +\sum_{j=1}^{k}x_{j}\beta_{j}+\epsilon_{i}$ represent the actual lactation yields. Then, we show that the regression coefficients calculated from estimated test-day yields, denoted by ${\hat{\beta }}_{j}^{*}$ , for test-day *j*, is the following, which do not correspond precisely to their “true” effects (denoted by $\beta_{j}$ ).

$${\hat{\beta }}_{j}^{*}=\frac{COV\left(x_{j}^{*},y\right)}{Var\left(x_{j}^{*}\right)}=\frac{COV\left(x_{j}+v_{j},x_{j}\beta_{j}\right)}{Var\left(x_{j}+v_{j}\right)}=\frac{\beta_{j}\sigma_{x_{j}}^{2}}{\sigma_{x_{j}}^{2}+\sigma_{v}^{2}}$$ (33)

Therefore, disregarding the errors linked to projected lactation milk yields from estimated test-day yields, instead of actual yields, does not imply their nonexistence. On the contrary, these errors give rise to biases known as regression dilution or regression attenuation (Frost and Thompson, 2000).

When measurement errors associated with test-day yields are significant, how do they affect the estimation of heritability, an important genetic parameter for genetic evaluations? Here, we give an analytical illustration based on a simplified animal model, where the overall mean ( μ ) is the only fixed effect and the random-effect terms include animal effects ( u ), namely, additive genetic effects, for test-day or lactation yields plus the residuals (***e***).

y=1μ+Zu+e (34)

Here, for example, y is a vector of daily milk yield directly measured on *n* cows on a given test day. The random animal effects $u∼N\left(0,A\sigma_{u}^{2}\right)$ are assumed to follow a multiple-variate normal distribution with null means and the variance-covariance matrix defined by the product of the numerator additive genetic relationship matrix A and a scalar quantity $\sigma_{u}^{2}$ . The residuals follow a multiple-normal distribution with null means and a common variance: $e∼N\left(0,I\sigma_{e}^{2}\right)$ .

The mixed-effect model equation (**MME**), in matrix form, is the following:

$$\left[\begin{array}{cc} 1^{\prime }1 & 1^{\prime }Z\\ Z^{\prime }1 & Z^{\prime }Z+A^{-1}\sigma_{u}^{-2} \end{array}\right]\left[\begin{array}{c} \hat{\mu }\\ \hat{u} \end{array}\right]=\left[\begin{array}{c} 1^{\prime }y\\ Z^{\prime }y \end{array}\right]$$ (35)

Following Henderson (1986), the estimated animal effects and residuals are the following:

$$\hat{u}=C_{2}W^{\prime }y$$ (36)

$$\hat{e}=y-1\hat{\mu }-Z\hat{u}$$ (37)

Here, $W=\left(\begin{array}{cc} 1 & Z \end{array}\right)$ , C and $C_{2}$ are given by taking the symmetric inverse of the coefficient matrix of (35), as follows:

$$C=\left[\begin{array}{c} C_{2}\\ C_{2} \end{array}\right]=\left[\begin{array}{cc} C_{11} & C_{12}\\ C_{21} & C_{22} \end{array}\right]={\left[\begin{array}{cc} 1^{\prime }1 & 1^{\prime }Z\\ Z^{\prime }1 & Z^{\prime }Z+A^{-1}\sigma_{u}^{-2} \end{array}\right]}^{-1}$$ (38)

In (37), $\hat{\mu }$ represents some solution for the overall mean, which is not unique when each animal has only one observation. Instead, we take a simple, parsimonious solution, which is the arithmetic average of daily milk yields:

$$\hat{\mu }=\frac{1}{n}1^{\prime }y$$ (39)

The estimated variances for the random genetic effects and residuals are the following (Henderson, 1986):

$${\hat{\sigma }}_{u}^{2}=\frac{1}{q}\left({\hat{u}}^{\prime }A^{-1}\hat{u}+tr\left(A^{-1}C_{22}\right)\right)$$ (40)

$${\hat{\sigma }}_{e}^{2}=\frac{1}{n}\left({\hat{e}}^{\prime }\hat{e}+tr\left(\mathrm{WC}W^{\prime }\right)\right)$$ (41)

where q is the number of random effects, and n is the number of observations.

Then, the heritability for this test-day milk yield is estimated as follows:

$$h^{2}=\frac{\sigma_{u}^{2}}{\sigma_{u}^{2}+\sigma_{e}^{2}}=\frac{n\left({\hat{u}}^{\prime }A^{-1}\hat{u}+tr\left(A^{-1}C_{22}\right)\right)}{n\left({\hat{u}}^{\prime }A^{-1}\hat{u}+tr\left(A^{-1}C_{22}\right)\right)+q\left({\hat{e}}^{\prime }\hat{e}+tr\left(\text{WC}W^{\prime }\right)\right)}$$ (42)

When daily milk yield is not measured directly but estimated from partial yields, the estimated daily yields may not precisely correspond to the actual test-day milk yields. Let ϵ be a vector of the deviates of estimated daily milk yields ( $y^{*}$ ) from their actual values, that is,

$$y^{*}=y+\epsilon$$ (43)

Then, the animal model becomes:

$$y^{*}=1\mu^{*}+Zu^{*}+e^{*}$$ (44)

In the above, we used * to distinguish between variables in the model (44) from those in the model (34). Then, we show that the overall mean, when obtained as the arithmetic average of corrected test-day milk yields, remains the same as in (39):

$${\hat{\mu }}^{*}=\frac{1}{n}1^{\prime }y^{*}=\frac{1}{n}1^{\prime }\left(y+\epsilon \right)=\frac{1}{n}1^{\prime }y+\frac{1}{n}1^{\prime }\epsilon =\frac{1}{n}1^{\prime }y$$ (45)

The above holds because $E\left(\epsilon \right)=\frac{1}{n}1^{\prime }\epsilon =0$ . However, the estimated random animal effects and residuals appear different. That is,

$${\hat{u}}^{*}=C_{2}W^{\prime }y^{*}=C_{2}W^{\prime }\left(y+\epsilon \right)=C_{2}W^{\prime }y+C_{2}W^{\prime }\epsilon =\hat{u}+C_{2}W^{\prime }\epsilon$$ (46)

$${\hat{e}}^{*}=y^{*}-1{\hat{\mu }}^{*}-Z{\hat{u}}^{*}=\left(y+\epsilon \right)-1\hat{\mu }-Z\left(\hat{u}+C_{2}W^{\prime }\epsilon \right)=\hat{e}+\left(\epsilon -{\text{ZC}}_{2}W^{\prime }\epsilon \right)$$ (47)

Assume that deviates happen randomly such that $E\left(\epsilon \right)=0$ . Then, we have $E\left({\hat{u}}^{*}\right)=E\left(\hat{u}\right)$ and $E\left({\hat{e}}^{*}\right)=E\left(\hat{e}\right)$ . However, when systematic biases are present, the bias increases as the milking interval becomes more uneven (Wu et al., 2022). Even when the estimated breeding values are unbiased, as in the case of random deviations, the variance components obtained from corrected test-day milk yields do not correspond precisely to those obtained from actual test-day milk yields. This is analytically shown as follows:

$${\hat{\sigma }}_{u^{*}}^{2}=\frac{1}{q}\left({\hat{u}}^{{*}^{\prime }}A^{-1}{\hat{u}}^{*}+tr\left(A^{-1}C_{22}\right)\right)=\frac{1}{q}\left({\left(\hat{u}+C_{2}W^{\prime }\epsilon \right)}^{\prime }A^{-1}\left(\hat{u}+C_{2}W^{\prime }\epsilon \right)+tr\left(A^{-1}C_{22}\right)\right)$$

Letting $b=C_{2}W^{\prime }\epsilon$ , which quantifies the deviation of ${\hat{u}}^{*}$ from ${\hat{u}}^{*}$ , the above becomes:

$${\hat{\sigma }}_{u^{*}}^{2}=\frac{1}{q}\left({\left(\hat{u}+b\right)}^{\prime }A^{-1}\left(\hat{u}+b\right)+tr\left(A^{-1}C_{22}\right)\right)=\frac{1}{q}\left(\hat{u}{\prime A}^{-1}\hat{u}+2{\hat{u}}^{\prime }A^{-1}b+b^{\prime }A^{-1}b+tr\left(A^{-1}C_{22}\right)\right)=\sigma_{u}^{2}+\frac{1}{q}\left(2{\hat{u}}^{\prime }A^{-1}b+b^{\prime }A^{-1}b\right)$$ (48)

Following a similar strategy and letting $d=\epsilon -{\mathrm{ZC}}_{2}W^{\prime }\epsilon$ , we have:

$${\hat{\sigma }}_{e^{*}}^{2}=\frac{1}{n}\left({\left(\hat{e}+d\right)}^{\prime }\left(\hat{e}+d\right)+tr\left(\text{WC}W^{\prime }\right)\right)=\frac{1}{n}\left({\hat{e}}^{\prime }\hat{e}+2{\hat{e}}^{\prime }d+d^{\prime }d+tr\left(\text{WC}W^{\prime }\right)\right)=\sigma_{e}^{2}+\frac{1}{n}\left(2{\hat{e}}^{\prime }d+d^{\prime }d\right)$$ (49)

Consequently, the heritability estimate obtained from estimated test-day milk yields also deviates from the heritability of actual daily milk yields. The following adjustments are needed to retain the same heritability as (42).

$$h^{2}=\frac{\sigma_{u^{*}}^{2}-\frac{1}{q}\left(2{\hat{u}}^{\prime }A^{-1}b+b^{\prime }A^{-1}b\right)}{\left(\sigma_{u^{*}}^{2}+\sigma_{e^{*}}^{2}\right)-\left(\frac{1}{q}\left(2{\hat{u}}^{\prime }A^{-1}b+b^{\prime }A^{-1}b\right)+\frac{1}{n}\left(2{\hat{e}}^{\prime }d+d^{\prime }d\right)\right)}=\frac{\sigma_{u}^{2}}{\left(\sigma_{u}^{2}+\sigma_{e}^{2}\right)}$$ (50)

Otherwise, if large in magnitude, the estimation errors associated with test-day milk yields would have significant implications, potentially influencing heritability estimates and the estimated breeding values. A thorough understanding of this situation hinges on further studies.

## Conclusion

We provided a comprehensive review of test-day milk yields, focusing on two primary daily yield correction methods and their applications, drawing insights from seminal studies over the past century. Test-day records offer meticulous insights into a cow’s productive life, elucidating the intricate interplay of genetic and environmental determinants shaping the quantity and quality of milk production. Such information is pivotal in formulating advanced mathematical models, thereby refining milk yield projections. The incorporation of test-day milk yields in dairy cattle genetic evaluations is instrumental in establishing a basis for heritability assessment and breeding value determination. This, in turn, steers breeding strategies aimed at elevating future milk yields and overall herd efficacy. Moreover, these yield records furnish invaluable perspectives for informed dairy management decisions, from fine-tuning feed efficiency and fortifying animal wellbeing to strategic culling decisions. This paper deliberates on the ramifications of modulating test-day milk yields, highlighting possible challenges and their potential effects on ensuing genetic evaluations.

The consistent acquisition and nuanced analysis of test-day data remain central to the progressive, sustainable, and profitable evolution of the dairy industry. In an ongoing endeavor, extensive, high-resolution milking data are being gathered for subsequent research. This initiative receives joint support from the US Council on Dairy Cattle Breeding, the USDA Agricultural Genomics and Improvement Laboratory, and the National Dairy Herd Information Association. Access to this new dataset will enable a thorough re-evaluation of existing systems and the development of novel tools, ensuring they reflect the most current and relevant realities. It will also facilitate the updating of pertinent parameters. Follow-up projects will include the reassessment of corrections applied to lactation yields and the refinement of adjustments for mature equivalent yields. These are crucial aspects for the future planning and enhancement of genetic evaluations and dairy production systems in the United States. As scientific and technological capabilities advance, we anticipate a heightened utilization of milk yield records, promoting more efficient genetic selection and herd management practices, thereby driving the dairy sector towards a profoundly knowledge-based and data-informed future.
